# Effects of oral butyrate supplementation on inflammatory potential of circulating peripheral blood mononuclear cells in healthy and obese males

**DOI:** 10.1038/s41598-018-37246-7

**Published:** 2019-01-28

**Authors:** Maartje C. P. Cleophas, Jacqueline M. Ratter, Siroon Bekkering, Jessica Quintin, Kiki Schraa, Erik S. Stroes, Mihai G. Netea, Leo A. B. Joosten

**Affiliations:** 1 Department of Internal Medicine, Radboud university medical center, Nijmegen, The Netherlands; 2 Radboud Institute for Molecular Life Sciences (RIMLS), Radboud university medical center, Nijmegen, The Netherlands; 3 Nutrition, Metabolism and Genomics Group, Division of Human Nutrition, Wageningen University, Wageningen, The Netherlands; 4 Department of Vascular Medicine, Academic Medical Center, University of Amsterdam, Amsterdam, The Netherlands; 5 Immunology of Fungal Infections group, Department of Mycology, Institut Pasteur, Paris, France; 6 Department for Genomics & Immunoregulation, Life and Medical Sciences Institute (LIMES), University of Bonn, 53115 Bonn, Germany; 7 Department of Medical Genetics, Iuliu Haţieganu University of Medicine and Pharmacy, Cluj-Napoca, Romania

## Abstract

Sodium butyrate is well-known for its immune-modulatory properties. Studies until now only focused on the *in vitro* effects of butyrate or assessed local effects in the gut upon butyrate administration. In this trial, we studied the systemic anti-inflammatory effects induced by sodium butyrate supplementation in humans. Nine healthy (Lean) and ten obese (metabolic syndrome group, MetSyn) males were given 4 grams sodium butyrate daily for 4 weeks. PBMCs were isolated before and after supplementation for direct stimulation experiments and induction of trained immunity by oxidized low-density lipoprotein (oxLDL), β-glucan, or Bacillus Calmette-Guérin vaccine (BCG). Butyrate supplementation moderately affected some of the cytokine responses in the MetSyn group. In the direct stimulation setup, effects of butyrate supplementation were limited. Interestingly, butyrate supplementation decreased oxLDL-induced trained immunity in the MetSyn group for LPS-induced IL-6 responses and Pam3CSK4-induced TNF-α responses. Induction of trained immunity by β-glucan was decreased by butyrate in the MetSyn group for Pam3CSK4-induced IL-10 production. In this study, while having only limited effects on the direct stimulation of cytokine production, butyrate supplementation significantly affected trained immunity in monocytes of obese individuals with metabolic complications. Therefore, oral butyrate supplementation may be beneficial in reducing the overall inflammatory status of circulating monocytes in patients with metabolic syndrome.

## Introduction

The sophisticated symbiosis between humans and colonizing microbes in the gut has been studied intensively during the last decades. Extensive research in the last years has shown that the microbial community living in our gut plays a significant role in maintaining our health. Its main functions include harvesting energy from dietary components, maintaining the intestinal mucosal barrier, and regulating immune responses^1,2^. One important microbial process that has been linked to all of these functions is represented by the production of short-chain fatty acids (SCFAs), a biological process taking place in the large intestine^3^.

For the digestion of the complex carbohydrates mainly found in fruit, vegetables and whole grains, we rely on fermentative metabolism by a group of anaerobic gram-positive bacteria in the colon. This process yields energy and SCFAs; mainly acetate, propionate and butyrate. Despite the fact that these metabolites consist of only a few carbon atoms, they have been shown to play a substantial role in health and disease. Butyrate in particular not only constitutes the main source of energy for colonocytes^4,5^, it has also been the focus of research due to its potent immune-regulating effects. Many studies report inhibition of pro-inflammatory cytokines, NF-κB activation, and nitric oxide production by butyrate *in vitro*, although the exact effects appear to depend on cell type, stimulus and concentration of butyrate^6–10^.

Two main mechanisms have been suggested to mediate the effects of butyrate. Firstly, butyrate was reported to be a broad histone deacetylase (HDAC) inhibitor, inhibiting most isoforms of class I and II^11–14^. This leads to hyperacetylation of both histone and non-histone proteins such as transcription factors, altering gene expression patterns. Secondly, butyrate binds G protein-coupled receptors 41 and 43, which were later renamed to fatty acid receptors FFA3 and FFA2, respectively. FFA2 is found predominantly on peripheral blood mononuclear cells (PBMCs), monocytes, and neutrophils^15,16^, but its exact role in regulating the immune system is still a topic of debate. Both protective and detrimental effects of FFA2 are reported in murine models of inflammatory diseases^17,18^. In addition, it is unknown whether FFA2 is able to directly affect cytokine production or which pathways are involved in this.

The concentrations of butyrate required for HDAC inhibition and FFA2/FFA3 activation both exceed physiological levels in the peripheral blood, but are well within range of butyrate concentrations in the gut^12,15,19^. We therefore envisage that monocytes can encounter high concentrations of butyrate in the gut and change their inflammatory potential to mediate systemic effects. Previous studies in mice have shown that oral butyrate inhibits cytokine production in peritoneal macrophages^20^, protects from atherosclerotic lesions in the aorta^21^, prevents diet-induced obesity, and increases insulin sensitivity and mitochondrial function^22^. These results suggest that butyrate supplementation may be beneficial for the treatment of metabolic diseases in which exaggerated inflammation plays an important role such as diabetes, atherosclerosis and gout.

Although oral butyrate supplementation in humans has been shown to ameliorate clinical symptoms of inflammatory bowel disease^23,24^, data on its effects on the inflammatory potential of circulating monocytes are lacking. In the present pilot study, healthy and obese subjects received 4 grams of butyrate daily for 4 weeks. We assessed the effects of butyrate supplementation on the capacity of circulating monocytes to produce innate immune cytokines directly in response to a range of endogenous and pathogenic stimuli. Furthermore, we investigated whether oral butyrate influences the capacity of monocytes to be trained by oxLDL or microbial components to respond more vigorously to a secondary unrelated stimulus, a process described previously as trained innate immunity^25–27^.

## Results

### Baseline characteristics

Ten lean males and ten obese males were included in the analysis. At baseline, the obese (MetSyn) group was characterized by a higher age, BMI, diastolic blood pressure, fasting plasma glucose, plasma low-density lipoprotein cholesterol, plasma triglycerides, and neutrophils counts, as well as lower plasma high-density lipoprotein cholesterol (Table 1). General data and metabolic syndrome parameters in Table 1 have also been published as baseline characteristics in a previous publication on the same population (Table 1 in^28^). There is a slight difference with the numbers in the previously published table due to the exclusion of one donor in the other paper^28^.

**Table 1:** Baseline characteristics.

	Lean group	MetSyn group
**General**		
n	**10**	**10**
Age	25 ± 2.4	42 ± 2.4***
**Metabolic syndrome parameters**		
Body mass index (kg/m^2^)	22.0 ± 2.3	33.2 ± 3.6***
Systolic blood pressure (mmHg)	130 ± 9	139 ± 16
Diastolic blood pressure (mmHg)	75 ± 6	82 ± 6*
Fasting plasma glucose (mmol/L)	4.4 ± 0.3	4.9 ± 0.4*
**Circulating lipids**		
Total cholesterol (mmol/L)	4.2 ± 0.8	4.9 ± 0.7
High-density lipoprotein cholesterol (mmol/L)	1.4 ± 0.2	1.0 ± 0.2**
Low-density lipoprotein cholesterol (mmol/L)	2.3 ± 0.6	2.8 ± 0.4*
Triglycerides (mmol/L)	0.8 ± 0.3	1.8 ± 0.3***
**Blood cell differentiation**		
Neutrophil count (×10^9^/L)	2.49 ± 0.70	3.23 ± 0.31*
Lymphocyte count (×10^9^/L)	1.94 ± 0.65	1.92 ± 0.52
Monocyte count (×10^9^/L)	0.48 ± 0.09	0.56 ± 0.12

At baseline, various parameters were measured to define the two subject groups. Statistically significant differences between the two subject groups are represented as follows: *p < 0.05, **p < 0.001, ***p < 0.0001. Data are presented as mean ± standard deviation. Part of data from this table is also published in^28^.

### Short-chain fatty acid concentrations in plasma and feces

The effects of 4 weeks oral sodium butyrate supplementation were assessed by measuring SCFA levels in the plasma and feces and were compared between volunteers in the Lean and MetSyn group. At baseline, the MetSyn group showed a different composition of plasma SCFA, with a lower percentage of acetate and higher percentages of propionate and butyrate (Table 2) compared to the Lean group. There were no baseline differences in fecal SCFA concentrations between the two groups (Table 3). In contrast, butyrate supplementation affected the fecal SCFA concentrations to a greater extent than plasma levels. Only the plasma propionate concentration was decreased in the MetSyn group after treatment compared to baseline. In feces, total SCFA, acetate, and propionate concentrations were significantly lower after 4-week butyrate supplementation in both the Lean and the MetSyn group. Fecal butyrate concentrations were only significantly decreased after butyrate supplementation in the MetSyn group.

**Table 2:** SCFA concentrations in plasma before and after oral butyrate supplementation.

Group	Time point	Total SCFA (µM)	Acetate (µM)	Acetate (%)	Propionate (µM)	Propionate (%)	Butyrate (µM)	Butyrate (%)
Lean	0	112.3 ± 45.5	101.1 ± 43.3	89.5 ± 3.4	8.1 ± 3.3	7.6 ± 2.9	3.1 ± 0.9	2.8 ± 0.7
Lean	4 w	114.2 ± 38.3	103.0 ± 34.2	90.4 ± 2.7	7.0 ± 3.7	6.1 ± 2.3	4.3 ± 3.4	3.5 ± 1.8
MetSyn	0	79.9 ± 23.1	68.6 ± 20.7	85.6 ± 2.8*	8.2 ± 2.7	10.4 ± 2.8*	3.2 ± 1.0	4.0 ± 0.8**
MetSyn	4 w	67.7 ± 28.2	59.0 ± 25.5	86.9 ± 5.0	5.6 ± 2.6^#^	8.6 ± 3.9	3.1 ± 2.8	4.5 ± 3.2

Short chain fatty acid (SCFA) concentrations in plasma were measured before and after butyrate supplementation in healthy lean males (Lean) and obese males (MetSyn). *Represents a statistically significant difference at baseline between the Lean and MetSyn groups, *p < 0.05 **p < 0.01. ^#^Represents a statistically significant difference between time point 0 and time point 4 w (4 weeks), p < 0.05. Data are presented as mean ± standard deviation.

**Table 3:** SCFA concentrations in feces before and after oral butyrate supplementation.

Group	Time point	Total SCFA (µmol/g)	Acetate (µmol/g)	Acetate (%)	Propionate (µmol/g)	Propionate (%)	Butyrate (µmol/g)	Butyrate (%)
Lean	0	344.2 ± 220.6	213.6 ± 146.1	61.7 ± 3.8	73.0 ± 61.9	20.1 ± 4.1	57.6 ± 29.4	18.2 ± 5.6
Lean	4 w	186.1 ± 79.8^#^	114.4 ± 49.5^#^	62.1 ± 10.2	41.0 ± 22.0^#^	21.6 ± 4.5	30.7 ± 25.0	16.3 ± 8.0
MetSyn	0	340.6 ± 141.9	208.3 ± 97.6	60.1 ± 7.9	78.3 ± 36.9	23.8 ± 6.8	54.0 ± 23.9	16.2 ± 4.1
MetSyn	4 w	226.9 ± 129.0^##^	139.8 ± 85.3^#^	61.9 ± 9.7	44.5 ± 25.9^##^	20.3 ± 5.9	42.5 ± 34.8^#^	17.9 ± 8.5

Short chain fatty acid (SCFA) concentrations in feces were measured before and after butyrate supplementation in healthy lean males (Lean) and obese males (MetSyn). ^#^Represents a statistically significant difference between time point 0 and time point 4 w (4 weeks), ^#^p < 0.05 ^##^p < 0.01. Data are presented as mean ± standard deviation.

### Effects of sodium butyrate treatment on total blood cell counts

To study the possible effects of sodium butyrate supplementation on white blood cell counts, blood differential tests were performed before and after butyrate supplementation. As mentioned above, only neutrophil counts differed significantly at baseline between the lean and obese groups (Table 1). No differences were found in percentages or absolute counts of neutrophils, lymphocytes, or monocytes in whole blood between the time points before and after supplementation (Supplemental Fig. 1).

### Cytokine production of circulating peripheral blood mononuclear cells

**Figure 1:**
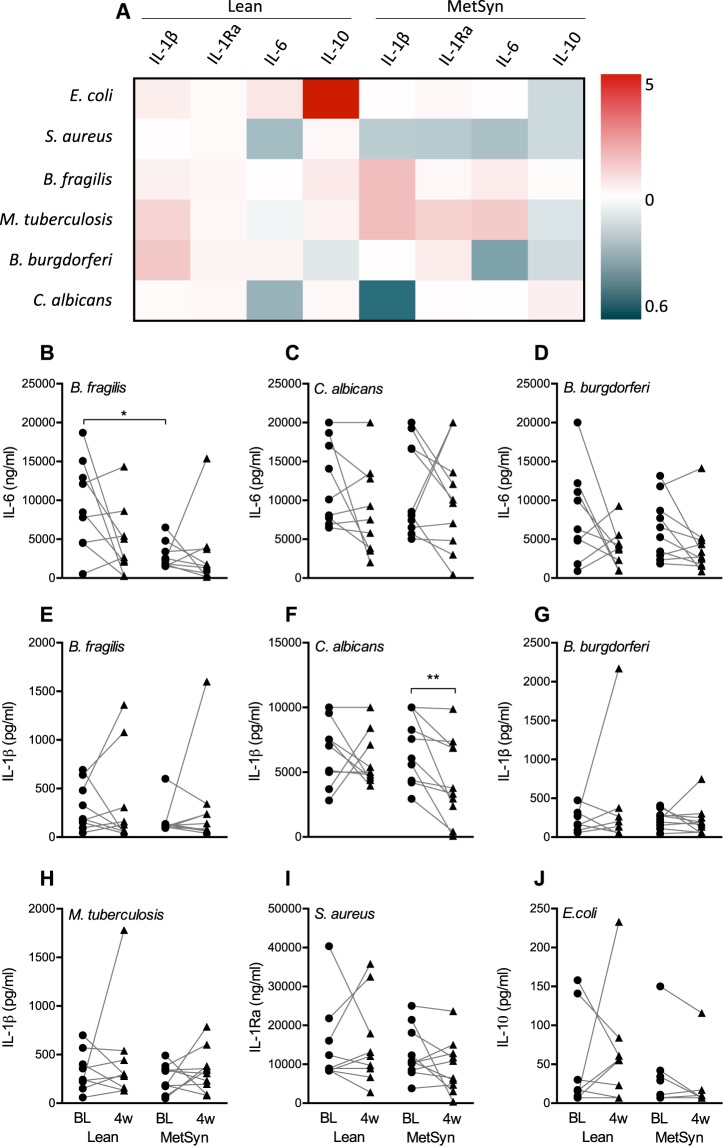
Effects of 4-week butyrate supplementation on PBMC cytokine responses to pathogens. Freshly isolated PBMCs from healthy lean volunteers (Lean) and metabolic syndrome patients (MetSyn) were cultured for 24 hours in the presence of pathogens before and after 4-week supplementation with 4 grams oral butyrate daily. (**A**) A heatmap shows the mean fold decrease or increase in cytokine production after supplementation in both groups. (**B**–**J**) Stimuli-cytokine combinations showing a substantial decrease or increase in panel A are highlighted in separate graphs to show the absolute cytokine values at baseline (BL) and 4 weeks (4 w). Lean n = 9, MetSyn n = 10.

To assess the effects of oral sodium butyrate supplementation on the cytokine-producing capacity of PBMCs, we stimulated the cells with a range of heat-killed human pathogens. An overall glance on the heatmap (Fig. 1A) representing the mean fold increase or decrease in cytokine production after butyrate supplementation, shows that the differences are mostly small. The following panels in Fig. 1 show the absolute cytokine production of stimuli-cytokine combinations that have a statistically significant effect or show larger effects in the heatmap. Baseline comparison between the groups showed that PBMCs from MetSyn volunteers were only less capable of mounting an IL-6 response to stimulation with *Bacteroides fragilis* compared to volunteers in the Lean group (Fig. 1B). After 4 weeks of oral butyrate supplementation, PBMCs from MetSyn subjects produced less IL-1β in response to *Candida albicans* (Fig. 1F) compared to baseline. As shown by the IL-10 production in response to *Escheria coli* (Fig. 1A,J), there is sometimes large donor variation, causing the heatmap to show somewhat biased differences.

**Figure 2:**
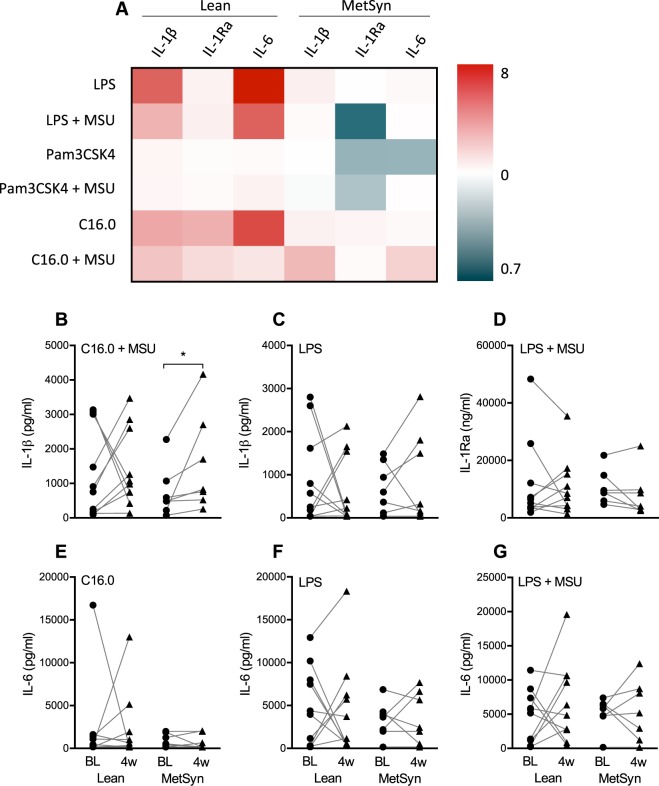
Effects of 4-week butyrate supplementation on PBMC cytokine responses to monosodium urate crystals. Freshly isolated PBMCs from healthy lean volunteers (Lean) and metabolic syndrome patients (MetSyn) were cultured for 24 hours in the presence of monosodium urate crystals in combination with inflammatory stimuli before and after 4-week supplementation with 4 grams oral butyrate daily. (**A**) A heatmap shows the mean fold decrease or increase in cytokine production after supplementation in both groups. (**B**–**G**) Stimuli-cytokine combinations showing a substantial decrease or increase in panel A are highlighted in separate graphs to show the absolute cytokine values at baseline (BL) and 4 weeks (4 w). Lean n = 10, MetSyn n = 7.

Because gouty arthritis is highly associated with metabolic syndrome, PBMCs were also stimulated with a combination of monosodium urate crystals (MSU) and Toll-like receptor ligands to induce a potent cytokine response. No differences in cytokine production were observed between the Lean and MetSyn groups at baseline (Fig. 2). The heatmap even shows mainly increases in IL-1β and IL-6, and decreases in the anti-inflammatory cytokine IL-1Ra (Fig. 2A). Surprisingly, the IL-1β production in response to a combination of MSU crystals and palmitic acid (C16.0) was significantly increased in the MetSyn group after oral butyrate supplementation (Fig. 2B).

### Training capacity of innate immune cells

One additional feature of monocytes is their capacity to be trained to produce more cytokines upon secondary stimulation with an unrelated stimulus^29^. This process is mediated through epigenetic reprogramming and metabolic rewiring of the macrophages and can, among others, be achieved by oxidized low-density lipoprotein (oxLDL), β-glucan, or Bacillus Calmette-Guérin vaccine (BCG)^25–27,30^.

**Figure 3:**
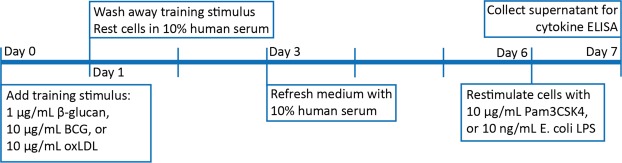
*In vitro* innate immune training setup. In freshly isolated adherent monocytes from healthy lean volunteers (Lean) and metabolic syndrome patients (MetSyn) training was induced by 24 hour culture with β-glucan, Bacillus Calmette-Guérin vaccine (BCG) or oxidized low-density lipoprotein (oxLDL). The training stimulus was washed out with warm phosphate-buffered saline and cells were rested for 5 days in medium with 10% pooled human serum. On day 6, cells were restimulated with 10 µg/mL Pam3CSK4 or 10 ng/mL *E. coli* lipopolysaccharide (LPS) for 24 hours, after which supernatant was collected for cytokine ELISAs.

**Figure 4:**
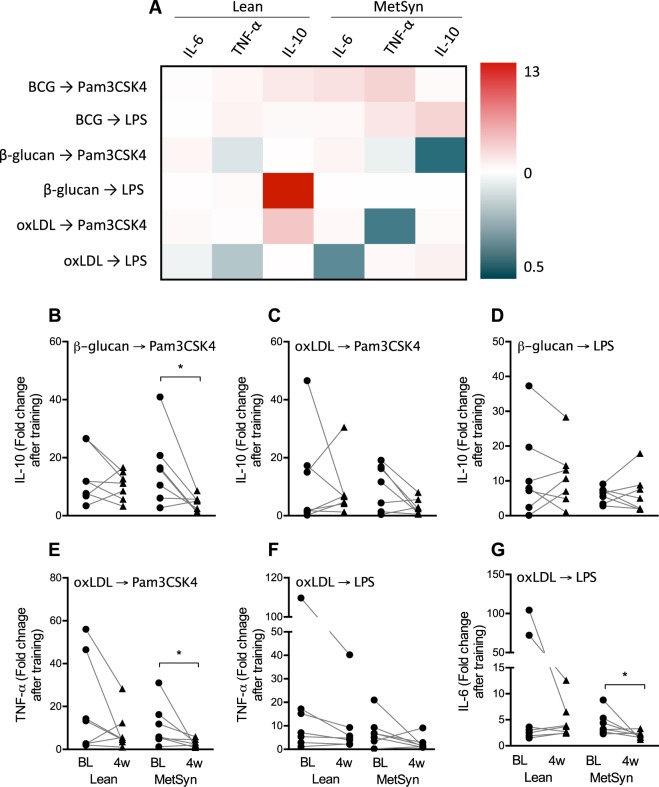
Effects of 4-week butyrate supplementation on monocyte training capacity. In freshly isolated adherent monocytes from healthy lean volunteers (Lean) and metabolic syndrome patients (MetSyn) training was induced as depicted in Fig. 3. This experiment was performed before and after 4-week supplementation with 4 grams oral butyrate daily. (**A**) A heatmap shows the mean fold decrease or increase in cytokine production with training after supplementation in both groups. (**B**–**G**) Stimuli-cytokine combinations showing a substantial decrease or increase in panel A are highlighted in separate graphs to show the fold change with training at baseline (BL) and 4 weeks (4 w). Lean n = 7, MetSyn n = 7.

**Figure 5:**
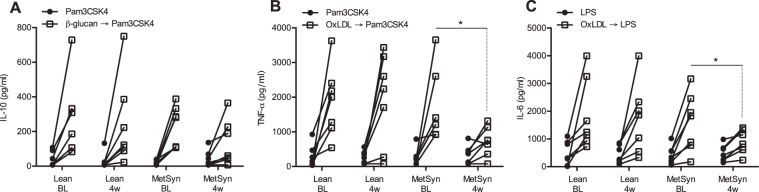
Effects of 4-week butyrate supplementation on absolute cytokine production in trained monocytes. This figure depicts the absolute cytokine responses in trained monocytes of those experimental setups in Fig. 4 that were significantly affected by butyrate supplementation. (**A**) shows the IL-10 responses in both groups at baseline (BL) and 4 weeks (4 w) after Pam3CSK4 stimulation with and without β-glucan training. (**B**) shows the absolute TNF-α responses in both groups after Pam3CSK4 stimulation with and without oxLDL training. (**C**) shows the IL-6 responses in both groups after LPS stimulation with and without oxLDL training. Time points within one group of volunteers were compared with a Mann-Whitney U-test. Lean n = 7, MetSyn n = 7.

To study the effect of butyrate supplementation on this process, we measured the training capacity of adherent monocytes before and after the 4-week supplementation according to the schedule in Fig. 3. The heatmap (Fig. 4A) shows a few decreases after butyrate production in the MetSyn group. The three strongest specific comparisons (Fig. 4B,E and G) yield statistical significance. After 4 weeks of butyrate supplementation, training induced by β-glucan upon restimulation with Pam3CSK4 was decreased for IL-10 production in the MetSyn group (Fig. 4B). Additionally, monocytes isolated from volunteers in the MetSyn group displayed a decreased training capacity by oxLDL after butyrate supplementation. Upon restimulation with Pam3CSK4, the fold increase in TNF-α production was decreased compared to baseline (Fig. 4E), and upon restimulation with LPS there was a significantly reduced up-regulation of IL-6 (Fig. 4G). There were no differences in baseline training capacity between the Lean and the MetSyn group. In Fig. 5, we show the absolute cytokine responses of the training experiments in which butyrate supplementation had a statistically significant effect. When looking at absolute cytokine levels instead of fold change with training, we observe no significant effects in the IL-10 production after β-glucan training and Pam3CSK4 restimulation (Fig. 5A). However, the TNF-α production upon training with oxLDL and restimulation with Pam3CSK4 is significantly lower compared to the absolute cytokine levels before butyrate supplementation (Fig. 5B). The same is observed with IL-6 production after training with oxLDL and restimulation with LPS (Fig. 5C).

## Discussion

In the present study we assessed the effects of 4 weeks oral sodium butyrate supplementation on the inflammatory potential of PBMCs in healthy lean and obese male human volunteers. To our knowledge this is the first clinical trial studying the influence of butyrate supplementation on systemic inflammatory responses in humans.

We show here that in comparison to the potent anti-inflammatory effects of butyrate observed predominantly in *in vitro* studies, the effects of oral butyrate supplementation on *ex vivo* cytokine production by PBMCs in this study were limited. Direct stimulation of PBMCs resulted in decreased IL-1β production upon stimulation with *Candida albicans* conidia, but increased C16.0 + MSU-induced IL-1β after butyrate supplementation in the MetSyn group. This is in contrast to the direct and potent anti-inflammatory effect of butyrate on C16.0 + MSU-induced *in vitro* cytokine production^12^. However, in the latter setup relatively high concentrations of butyrate were required to suppress histone deacetylases and the subsequent production of pro-inflammatory cytokines.

More consistent effects were found when assessing the impact of butyrate supplementation on the induction of trained innate immunity. Here, butyrate supplementation decreased oxLDL-induced trained immunity in the MetSyn group for LPS-induced IL-6 responses and Pam3CSK4-induced TNF-α responses. This effect was also seen when looking at absolute cytokine levels instead of training-induced fold increases. In addition, training with β-glucan was decreased for Pam3CSK4-induced IL-10 production. Interestingly, all effects observed after oral butyrate supplementation are limited to the MetSyn group. In the context of metabolic syndrome, especially oxLDL-induced trained innate immunity has been implicated to play a role in the development of atherosclerosis^31^. Short-term exposure of monocytes to oxLDL was shown to induce long-lasting epigenetic modifications that subsequently lead to a more pro-inflammatory macrophage phenotype^27^. Therefore, oral butyrate supplementation may be beneficial in preventing oxLDL-induced training of macrophages in patients with metabolic syndrome, possibly slowing down the process of vascular wall inflammation and the development of atherosclerosis.

Within the scope of the current study, we can only speculate on the mechanism of the effects of oral butyrate supplementation on systemic inflammatory responses. Our results suggest that the effects of oral butyrate supplementation are limited when looking at direct cytokine responses, but it may be able to inhibit the induction of trained innate immunity. From previous *in vitro* work we have seen that high concentrations of butyrate can induce inhibition of class I histone deacetylases, leading to inhibited cytokine production in PBMCs^12^. Possibly, butyrate concentrations after supplementation are not high enough, or its effects on histone deacetylases do not last long enough to inhibit cytokine responses upon direct stimulation of PBMCs.

The long-lasting effects of trained innate immunity are mediated through epigenetic reprogramming at the level of histone methylation^25^. Additionally, trained macrophages are characterized by a metabolic shift from mitochondrial oxidative phosphorylation to aerobic glycolysis that is mediated through mammalian target of rapamycin (mTOR) activation^26^. In contrast, butyrate has been shown to induce mitochondrial respiration and fatty acid oxidation in various *in vitro* and murine studies^4,22,32^. Based on this, it can be hypothesized that oral butyrate supplementation leads to high local concentrations of butyrate in the gut, where it can affect cellular metabolism of circulating monocytes, making them less susceptible to training. Furthermore, our observation that only the training capacity of PBMCs from obese males is affected by butyrate supplementation suggests there might be a role for microbiome or diet in this group, or that PBMCs from these subjects are more susceptible to the changes induced by butyrate.

The current study has several limitations. Firstly, this was a pilot study with small numbers in each group, providing only limited power to detect differences in cytokine production as these already show high variability among donors. Secondly, we were unable to detect any increases in butyrate concentration in the feces or plasma after 4 weeks of supplementation. Most likely, this is because butyrate is very quickly utilized as an energy source by colonocytes. With only an estimated 5% of SCFAs being excreted through feces^33^ and almost all of them present in circulation being metabolized by the liver^34^, neither plasma nor fecal SCFA concentration are a good measure of the transiently increased SCFA concentrations in the gut. In a recent paper, Canfora *et al*. shows that the increase in plasma butyrate concentration after colonic administration of sodium butyrate is very quick and already undetectable at 2 hours after colonic infusion^35^. The 4 weeks of butyrate supplementation did not lead to a stable increase in plasma SCFA levels, due to which we were unable to correlate changes in cytokine production directly to plasma SCFAs. After 4 weeks of butyrate supplementation we even detected decreases in plasma propionate, and fecal total SCFAs, acetate, propionate and butyrate. Within the scope of this study, we unfortunately have no method of exploring why this is occurring. Possibly, the addition of oral butyrate induced flux changes for acetate and propionate as well.

In conclusion, twice daily intake of 2 grams of sodium butyrate for 4 weeks most likely only transiently increased butyrate concentration in the gut. We found that this leads to a decreased training capacity of monocytes by oxLDL and β-glucan in the group of obese males. Oral butyrate supplementation may therefore be beneficial to reduce the overall inflammatory phenotype of circulating monocytes in metabolic syndrome patients and possibly even slow down the development of vascular wall inflammation and atherosclerosis. However, this will need to be studied in larger groups while closely looking at influences of the microbiome and diet on SCFA fluxes, and extensively examining vascular wall inflammation and atherosclerotic plaque formation.

### METHODS Study setup

The *ex vivo* experiments performed in this study are part of the larger OBUGAT study (Effect of Oral BUtyrate on human Glucose and brown fAT metabolism), which has been performed in the Academic Medical Center in Amsterdam, the Netherlands (personal correspondence with prof. Nieuwdorp). In this study, participants received oral butyrate supplementation of 4 grams daily for 4 weeks. Before and after treatment, venous EDTA blood was drawn for *ex vivo* experiments, and short-chain fatty acid concentrations were determined in plasma and feces. This study was approved by the medical research ethics committee of the Academic Medical Center in Amsterdam (METC number 2013_229) and conducted according to the principles in the Declaration of Helsinki. All volunteers gave informed consent. The study was registered in the WHO International Clinical Trial Registry Platform on 9 January 2014 under the reference number NTR4392.

### Subjects

In total 10 healthy lean males and 10 obese males were included in the study. To be eligible for inclusion in the study, subjects in the Lean group must be over 18 years of age and have a body mass index (BMI) of 19–25 kg/m^2^. Exclusion criteria were smoking, medication use, and an estimated glomerular filtration rate of <60. For inclusion in the obese (MetSyn) group, subjects must be over 18 years of age, have a body mass index (BMI) of >25 kg/m^2^, and possess 3 of the 5 criteria for metabolic syndrome. These have been described by the American Heart Association and National Heart, Lung, and Blood Institute as fasting plasma glucose ≥5.6 mmol/L, blood pressure ≥130/85 mmHg, triglycerides ≥1.69 mmol/L, high-density lipoprotein cholesterol <1.03 mmol/L, or a waist circumference ≥102 cm^36^. Exclusion criteria for this group are smoking and medication use.

### SCFA measurement in plasma and feces

Acetate, propionate and butyrate concentrations were determined in overnight fasted plasma samples and feces by liquid chromatography tandem mass spectrometry (LC-MS/MS) as described previously^37^. Feces were collected during 24 hours before study visits at baseline before butyrate supplementation and at 4 weeks after supplementation regime. Subjects kept the samples in the fridge at 3–4 °C before the visit and transported it on ice packs.

### PBMC isolation and direct stimulation

Peripheral blood mononuclear cells (PBMCs) were isolated using density gradient centrifugation with Ficoll-Paque and resuspended in Dutch modified RPMI culture medium (Invitrogen) supplemented with 50 µg/mL gentamycin (Centrafarm), 2 mM GlutaMAX and 1 mM pyruvate (Life Technologies). For the first stimulation experiments, the cells were cultured for 24 hours in the presence of 1 × 10^6^ CFU/mL heat-killed *Escherichia coli* (ATCC 35218, in house), 1 × 10^6^ CFU/mL *Staphylococcus aureus* (ATCC 29213, in house), 1 × 10^6^ CFU/mL *Bacteroides fragilis* (ATCC 25285, in house), 1 µg/mL *Mycobacterium tuberculosis* (H37Rv, in house), 1 × 10^6^ CFU/mL *Borrelia burgdorferi* (ATCC 35210), or 1 × 10^6^ *Candida albicans* conidia (UC820, in house). Secondly, in another experiment cells were cultured for 24 hours with 1 µg/mL Pam3CKS4 (EMC Microcollections), 1 ng/mL *E. coli* lipopolysaccharide (LPS, Sigma), or 50 µM palmitate (C16.0, Sigma, conjugated to human albumin as described in^12^) alone or in combination with monosodium urate (MSU) crystals (made in house with uric acid, Sigma, and sodium hydroxide, Merck, as described in^12^).

### Trained immunity experiments in adherent monocytes

For the training experiment, PBMCs were plated in a flat-bottom 96-well plate and left to adhere at 37 °C 5% CO_2_ for 1 hour, after which the cells were gently washed three times with warm phosphate-buffered saline (PBS). Subsequently, the cells were cultured for 24 hours with 1 µg/mL β-glucan (kindly provided by professor David Willliams, TN, USA), 10 µg/mL Bacillus Calmette-Guérin vaccine (BCG, Statens Serum Institute) or 10 µg/mL oxidized LDL (prepared in house as described before^38^). The cells were washed once with PBS at 37 °C and left to rest for 5 days, replenishing culture medium with 10% human pooled serum at day 3. At day 6, the cells were restimulated for 24 hours with 10 µg/mL Pam3CSK4 or 10 ng/mL *E. coli* LPS (see Fig. 3).

### Cytokine measurements and blood cell counts

After culturing the cells, supernatants were collected from the experiments. Interleukin-1β (IL-1β), interleukin-1 receptor antagonist (IL-1Ra), and tumor necrosis factor-α (TNF-α) concentrations in the supernatant were determined with commercially available ELISA kits from R&D Systems, and the ELISA kits for IL-6 and IL-10 were purchased from Sanquin. Blood differential test was performed with whole EDTA blood by the clinical diagnostic laboratory of the Radboudumc in Nijmegen, the Netherlands.

### Statistical analyses

The Lean and MetSyn groups of the study were compared using the Mann-Whitney U test and time points within one group were compared with the Wilcoxon signed rank test, both performed with Graphpad Prism software version 6.0. Due to the small group numbers in this pilot study no correction for multiple testing was applied. The number of subjects per group may differ for the different *ex vivo* experiments due to exclusion of donors giving a cytokine response to RPMI controls. Exact numbers per experiment are stated in the figure legends.

## Supplementary information

Approved trial protocol OBUGAT

Supplemental figure 1
