# Supplementary material for: Effects of oral butyrate supplementation on inflammatory potential of circulating peripheral blood mononuclear cells in healthy and obese males
Source: Sci Rep. 2019 Jan 28;9:775. doi: 10.1038/s41598-018-37246-7 (PMC6349871; doi:10.1038/s41598-018-37246-7)
Supplement: Supplementary file 1 — Approved trial protocol OBUGAT [file 41598_2018_37246_MOESM1_ESM.pdf]

Supplementary material

Effects of oral butyrate supplementation on inflammatory potential of circulating peripheral blood mononuclear cells in healthy and obese males

Maartje C.P. Cleophas[1,2], Jacqueline M. Ratter[1,2,3], Siroon Bekkering[1,2], Jessica Quintin[5], Kiki Schraa[1], Erik S. Stroes[4], Mihai G. Netea[1,2,6], Leo A.B. Joosten[1,2,7]

[1] Department of Internal Medicine, [2] Radboud Institute for Molecular Life Sciences (RIMLS), Radboud university medical center, Nijmegen, the Netherlands

[3] Nutrition, Metabolism and Genomics Group, Division of Human Nutrition, Wageningen University, Wageningen, the Netherlands

[4] Department of Vascular Medicine, Academic Medical Center, University of Amsterdam, Amsterdam, the Netherlands

[5] Immunology of Fungal Infections group, Department of Mycology, Institut Pasteur, Paris, France

[6] Department for Genomics & Immunoregulation, Life and Medical Sciences Institute (LIMES), University of Bonn, 53115 Bonn, Germany

[7] Department of Medical Genetics, Iuliu Haţieganu University of Medicine and Pharmacy, Cluj-Napoca, Romania

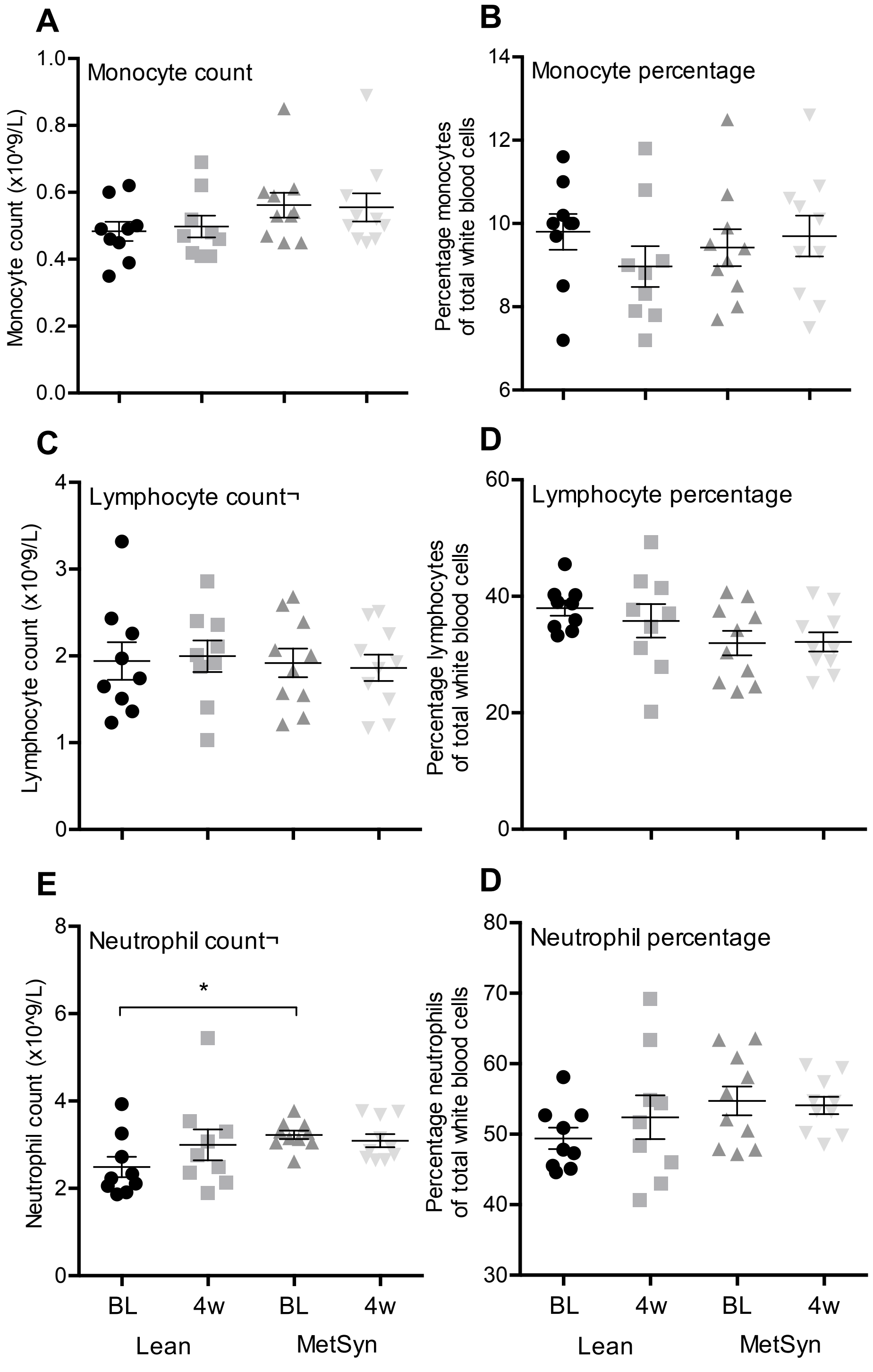

Supplemental Figure 1. Blood cell counts before and after butyrate supplementation Absolute counts and percentages of monocytes, lymphocytes and neutrophils were measured before and after butyrate supplementation. Baseline (BL) neutrophil counts were significantly higher in the MetSyn group compared to the lean individuals (panel E). No statistical significance was detected when comparing values before (BL) and after butyrate supplementation (4w).
